# Oral Treatments With the TrkB Ligand Prodrug, R13, Promote Enhanced Axon Regeneration Following Peripheral Nerve Injury

**DOI:** 10.3389/fncel.2022.857664

**Published:** 2022-04-15

**Authors:** Arthur W. English, Dario Carrasco, Dustin Hoffman, Robin Isaacson, Seong Su Kang, Samia Khan, Xia Liu, Keqiang Ye

**Affiliations:** ^1^Department of Cell Biology, Emory University School of Medicine, Atlanta, GA, United States; ^2^Rehabilitation Medicine, Emory University School of Medicine, Atlanta, GA, United States; ^3^Pathology and Laboratory Medicine, Emory University School of Medicine, Atlanta, GA, United States; ^4^Faculty of Life and Health Sciences, Brain Cognition and Brain Disease Institute, Shenzhen Institute of Advanced Technology, Chinese Academy of Sciences, Shenzhen, China

**Keywords:** BDNF (brain derived neurotrophic factor), nerve injuries, retrograde tracer, M response, small molecule

## Abstract

Axon regeneration after peripheral nerve injury is slow and inefficient, leading to generally poor functional recovery. Activity-dependent experimental therapies that increase expression of brain-derived neurotrophic factor (BDNF) and its TrkB receptors enhance regeneration, suggesting that treatments with BDNF might also be effective. However, recombinant human BDNF (rhBDNF), as well as 7,8-dihydroxyflavone (7,8-DHF), a small molecular BDNF mimetic, may have limited treatment applications because of their modest oral bioavailability and pharmacokinetic profile. R13 is a 7,8-DHF prodrug. Upon oral administration, it is converted in the liver to 7,8-DHF. In immunoblots from tissues at the site of nerve injury, a single oral treatment with R13 to mice following sciatic nerve transection and repair produced a rapid and prolonged increase in immunoreactivity to phosphorylated TrkB, prolonged phosphorylation of mitogen activated protein kinase (MAPK/Erk1/2), and a rapid but transient increase in phosphorylated AKT (protein kinase B). Intramuscular injections of fluorescent retrograde tracers into the gastrocnemius and tibialis anterior muscles 4 weeks after nerve injury resulted in significantly greater numbers of labeled motoneurons and dorsal root ganglion neurons in R13-treated mice than in vehicle-treated controls. Direct electromyographic (EMG) responses (M waves) were significantly larger in R13-treated mice 4 weeks after injury than vehicle-treated controls or mice treated with oral 7,8-DHF. Oral treatments with the prodrug, R13, are a potent therapy for stimulating axon regeneration and functional recovery after peripheral nerve injury.

## Introduction

Poor recovery from peripheral nerve injuries (PNIs) is a significant public health problem. These injuries are common and even though it is widely held that axons in injured nerves have greater regenerative potential than injured central nervous system axons, the process is slow and inefficient. The result is that only a very small proportion of individuals with injuries to peripheral nerves ever recover significant function (Portincasa et al., 2007; Scholz et al., 2009). Novel approaches to stimulating the regeneration of injured peripheral axons are needed to improve these poor outcomes and reduce the burden on affected individuals.

Among the most successful experimental therapies for PNI evaluated in preclinical models are application of low frequency (20 Hz) electrical stimulation (ES) and moderate treadmill exercise. Both have resulted in increased elongation of regenerating axons and reinnervation of their targets in the periphery (English et al., 2011; Gordon and English, 2016) and both have resulted in an increase in the number of neurons whose axons have regenerated successfully (Al-Majed et al., 2000; English et al., 2009). Both ES and exercise rely on an increase in the signaling between brain derived neurotrophic factor (BDNF) and its TrkB receptor in the regenerating axons (Gordon and English, 2016). Indeed, application of recombinant human BDNF (rhBDNF) to the repair site of a cut nerve in mice resulted in a marked enhancement of axon regeneration (Sabatier and English, 2015).

However, we sought alternate TrkB ligands because rhBDNF has a very short half-life in tissues. We identified two such small molecules, 7,8-dihydroxyflavone (7,8-DHF) (Jang et al., 2010b) and deoxygedunin (Jang et al., 2010a), and showed that their treatment, delivered either by local application to the injured nerve or by systemic injection, markedly enhanced axon regeneration in mouse models (English et al., 2013). Treatments with these small molecules did not simply promote the release of endogenous BDNF, as they enhanced regeneration in BDNF knockout mice. They did not promote axon regeneration in TrkB knockout mice, indicating that their success required intact TrkB receptors (English et al., 2013).

Despite the successful use of these small molecular TrkB ligands in enhancing axon regeneration, their translational potential has limitations. Local treatments with them are usually limited to singular surgical interventions. Stimulation of axon regeneration over longer distances, as is commonly necessary in human patients and which might require more than one application, would therefore be unlikely. In addition, the biological half-life of systemically applied 7,8-DHF is only slightly longer than that of rhBDNF (Chen et al., 2018), meaning that injections would have to be repeated often to be effective. For these reasons, we have examined the use of a prodrug, R13, that after oral administration is converted into 7,8-DHF by the liver (Chen et al., 2018). This approach significantly extends the duration of TrkB signaling and offers the advantage of oral dosing. The goal of this study was to evaluate the effectiveness of oral treatments with R13 on axon regeneration following peripheral nerve injury in a commonly used mouse model system.

## Materials and Methods

All experimental methods were approved by the Institutional Animal Care and Use Committee of Emory University (Protocol #PROTO201800101) and were consistent with the guidelines for Animal Research of the Society for Neuroscience. All animals used were C57B6 strain wild type (WT) mice. R13 was obtained from Sundia MediTech, Shanghai, China (Lot No: A0257-10014-16). It was dissolved in a 5% DMSO/0.5% methylcellulose vehicle solution.

### Site of Action Experiments

To evaluate whether oral R13 treatments resulted in TrkB activation at the nerve repair site, we used immunoblotting to evaluate the expression of phosphorylated TrkB (pTrkB Y705) and its effect on phosphorylation of the downstream effector molecules, AKT and MAPK/Erk1/2.

Animal treatment and sample preparation: In isoflurane-anesthetized 3-month-old WT mice of both sexes, we exposed the sciatic nerve in the mid-thigh and cut and repaired it by end-to-end anastomosis, as described in more detail below. Three days later, the mice were orally administered R13 (43.6 mg/Kg). This dose is equivalent to 30 mg/Kg of 7,8-DHF, and was chosen because it was the intermediate of three concentrations used to study the pharmacokinetics of R13 in brain and plasma (Chen et al., 2018). Other mice were given a similar volume of vehicle (5% DMSO/0.5% methylcellulose). Prior to (0 min) and at 15, 30, 60, 120, 240, and 480 min after R13/vehicle administration, mice were euthanized with Euthasol^®^ solution (sodium pentobarbital, 390 mg/mL) and the cut and repaired nerves were harvested, including 1 mm proximal and distal to the injury site.

Western blot analysis: The frozen nerves were lysed in Laemmli buffer (62.5 mM Tris-HCL, pH 6.8, 10% glycerol, 2% SDS, 5% 2-mercaptoethanol, 0.005% bromophenol blue) and followed by homogenization with sonication. After heating at 98°C for 5 min, similar amounts of tissue lysate from different nerves were separated by SDS-PAGE, transferred to nitrocellulose membranes, and probed with the antibodies listed in Table 1. Antibody binding was detected using appropriate peroxidase conjugated secondary antibodies and visualized using enhanced chemiluminescence.

**TABLE 1:** Antibodies used in immunoblots.

Antibody	Source	Dilution
pTrkB Y705	Cat# ab52191, Abcam	1:500
TrkB	Cat# 4603S, Cell Signaling	1:500
pAKT	Cat# 9271S, Cell Signaling	1:1,000
AKT	Cat# 4691s, Cell Signaling	1:2,000
pMAPK/ERK1/2	Cat# 9106S, Cell Signaling	1:1,000
MAPK/ERK1/2	Cat# 9102S, Cell Signaling	1:2,000
β-Actin	Cat# A1978, Sigma	1:10,000
Goat-anti-rabbit IgG HRP	Cat# 20–304, Genesee	1:2,000
Goat-anti-rabbit IgG HRP	Cat# 20–303, Genesee	1:2,000
For processing of immunoblots		
Product name		Source
Immobilon Forte western HRP substrate (ECL)		Cat# WBLUF050, Millipore

Quantification of intensity of immunoreactivity was performed using ImageJ. Values from blots probed for phosphorylated TrkB, AKT, and MAPK/Erk1/2 were compared to values of blots loaded with identical amounts of the same samples and probed for total TrkB, AKT, and MAP kinase. Ratios of phosphorylated to total protein in samples of nerves at different times after administration of R13 or vehicle were compared to the corresponding ratios obtained from mice prior to any administration of R13 or vehicle. Significance of differences in these ratios at different times after R13 administration, to that obtained prior to administration, was evaluated using unpaired *t*-tests.

### Electromyographic Studies

In young adult (6–8-week-old) mice anesthetized with isoflurane, the sciatic nerve was exposed in the mid-thigh and cut and repaired. The protocol for repair is similar to that described in more detail elsewhere (Akhter et al., 2019). Briefly, a small (1 × 2 mm) rectangle of medical grade silastic film was placed beneath the nerve and secured to it with one microliter of fibrin glue, a mixture of fibrinogen and thrombin. Once the glue had set, the nerve was cut near the center of the silastic mat and a second application of fibrin glue was applied to secure the anastomosed segments in place. Wounds were then closed in layers. Daily oral treatments with R13 (21.8 mg/Kg) (four male and four female mice), vehicle (four male and four female mice), or 7,8-DHF (15 mg/Kg) (four male and four female mice) were conducted 5 days per week for 2 weeks, beginning on the third day after injury. The timing of this treatment was designed to be similar to that used to treat animals with exercise (English et al., 2011). The dose of R13 used was the lowest effective dose reported by others. Based on their molecular weights, this dose of R13 is similar to a dose of 15 mg/Kg of 7,8-DHF (Chen et al., 2018).

To evaluate the extent of reinnervation of skeletal muscles by motor axons, direct muscle electromyographic (EMG) responses (M responses) were evoked in the gastrocnemius (GAST) and tibialis anterior (TA) muscles 4 weeks after injury, 2 weeks after the end of treatments with R13, 7,8-DHF, or vehicle. In isoflurane-anesthetized animals, the sciatic nerve was exposed as it exited the pelvis via short skin incision and two monopolar needle electrodes (Ambu #74325-36/40, Columbia, MD, United States) were placed next it and used to deliver electrical stimulus pulses to the nerve. Bipolar fine wire EMG electrodes (Basmajian and Stecko, 1963) were placed transcutaneously into the GAST and TA muscles using a 25G hypodermic needle. Once in place the wires were connected to differential amplifiers. Ongoing background EMG activity was sampled by a laboratory computer system running custom Labview^®^ software, and when activity fell within a user-defined resting level, the computer delivered a 0.3 ms duration constant voltage stimulus pulse to the needle electrodes. Evoked EMG activity, sampled at 10 KHz, was recorded, beginning from 20 ms prior to the stimulus and lasting until 50 ms after the stimulus. Stimulus intensity was increased gradually until a maximum amplitude M response (Mmax) was obtained. Stimuli were delivered no more frequently than once every 3 s to avoid fatigue.

Amplitudes of Mmax were measured off-line as the average full wave rectified voltage within the boundaries of the triphasic responses observed. In all the mice, these amplitudes were scaled to Mmax amplitudes recorded 4 weeks after sciatic nerve transection and repair in untreated animals, reported previously (Park et al., 2019). Scaled Mmax values were compared between the three treatment groups in the two muscles studied. The significance of differences in scaled M response amplitudes between groups was evaluated using ANOVA.

### Retrograde Labeling

Four R13-treated (two male and two female) and four vehicle-treated (two male and two female) 6–8-week-old mice were studied. Four weeks after bilateral sciatic nerve transection and repair and 2 weeks of daily oral administration of R13 or vehicle, as described above, retrograde fluorescent tracers were injected into the GAST and TA muscles. These would mark the cell bodies of motor and sensory neurons whose axons had regenerated and successfully reinnervated those muscle targets. In isoflurane-anesthetized mice, one microliter of a 1% solution of wheat germ agglutinin (WGA), conjugated either to Alexafluor 488 or Alexafluor 555, was injected into each of the medial and lateral heads of the GAST and into the TA muscle on both sides of the animals using a 35G needle attached to a 1 μl Hamilton syringe. The injections were made at two locations in each muscle/head and the needle was left in place for 5 min between injections to avoid leakage of the solutions. After the injections were completed the surgical field was flushed three times with sterile saline solution before the surgical wounds were closed and the mice were returned to their cages.

Three days after tracer injection, mice were euthanized with Euthasol^®^ solution and perfused transcardially with 4% paraformaldehyde solution, pH 6.9. Lumbar spinal cords and L4 dorsal root ganglia (DRGs) were harvested, post-fixed for 1 h, and cryoprotected overnight in 20% sucrose solution. Cryostat sections of spinal cords, at 40 μm thickness, were mounted onto charged slides and cover slipped using Vectashield^®^. Images of these sections at 20X magnification, using a Leica DM6000 upright fluorescence microscope and Hamamatsu low-light camera, were made using HCImage software. Labeled motoneurons were identified if the retrograde fluorescence filled the soma and extended into the proximal dendrites and if a clear area of the cell corresponding to the nucleus could be visualized, as we have described previously (English, 2005). Labeled profiles that did not meet these criteria were not counted. Counts of labeled motoneurons were made separately on the two sides of each spinal cord. Data from R13-treated mice were compared to those found in vehicle-treated animals.

Fourth lumbar (L4) dorsal root ganglia were sectioned on a cryostat at 40 μm thickness, mounted onto charged slides and cover slipped using Vectashield^®^. Imaging of these sections was identical to that used for spinal cords, above. A DRG neuron was considered labeled if the fluorescent marker filled the entire soma and a nuclear region could be identified. The soma cross sectional area of each labeled DRG neuron was measured using FIJI software. The relative numbers of small (<300 μm^2^), medium sized (300–700 μm^2^), and large (> 700 μm^2^) DRG neurons (Ruscheweyh et al., 2007) in the L4 DRGs of R13-treated and vehicle-treated were compared.

### Statistical Analyses

All statistical comparisons were performed using GraphPad Prism software. Alpha for significance of differences was set at *p* < 0.05 throughout.

## Results

### Oral R13 Treatment Induces TrkB Activation and Phosphorylation of Downstream Effectors at the Nerve Injury Site

**FIGURE 1:**
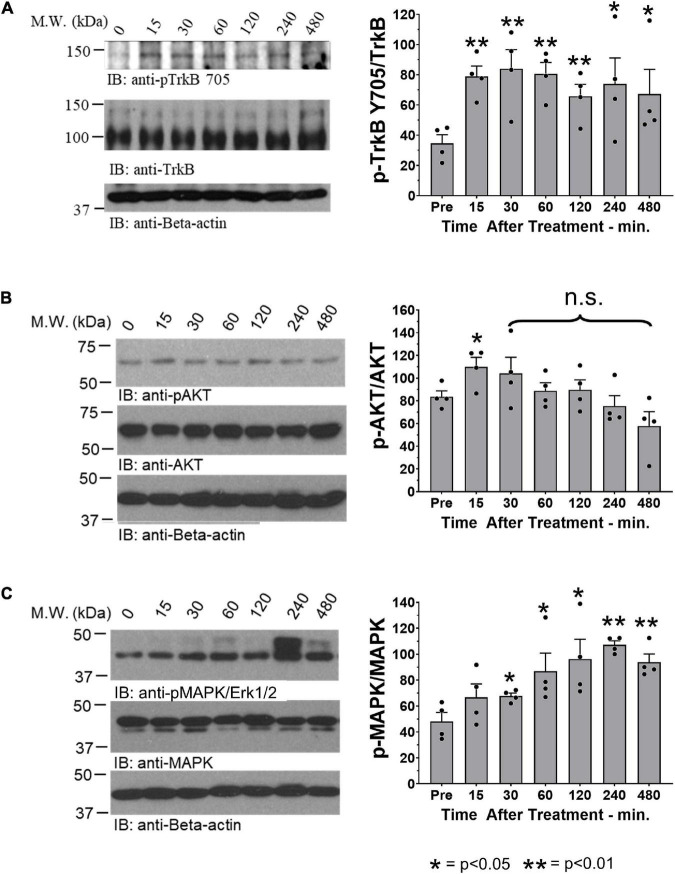
Oral R13 treatment stimulates TrkB phosphorylation and a downstream effector at the site of nerve injury. On the left side of each panel, examples of blots of sciatic nerves at the site of injury prior to (0 min) and at 15, 30, 60, 120, 240, and 480 min after oral administration of R13 are shown. On the right are quantitative results from blots from four nerves (*n* = 4). Bands of specific protein were densitometrically analyzed and normalized to the signal intensity of beta-actin. For each of the three proteins studied, the ratio of the expression of their activated (phosphorylated) form relative to total protein expression was calculated. Significance of differences at different times after administration from that prior to R13 administration was evaluated using an unpaired *t*-test: *p < 0.05 **p < 0.01, *n* = 4. **(A)** Immunoblots for anti- phosphorylated TrkB (pTrkB Y705), TrkB, and beta-actin. **(B)** Immunoblots for anti- phosphorylated AKT (pAKT), AKT, and beta-actin. **(C)** Immunoblots for anti- phosphorylated MAP kinase (pMAPK/Erk1/2), MAPK/Erk1/2, and beta-actin.

We have shown previously that oral treatment with R13 resulted in a prolonged metabolic production of 7,8-DHF and TrkB receptor activation (Chen et al., 2018). In the present experiments, we chose to investigate whether a similar treatment following peripheral nerve transection and repair would produce such effects *at the injury site*. We harvested cut and repaired sciatic nerves at different times after a single oral administration of R13 or vehicle, and analyzed the tissue for the presence of TrkB activation. In immunoblots we found a rapid increase in immunoreactivity, relative to pre-treatment levels (unpaired *t*-test, *t*_6_ = 4.937, *p* = 0.0013) to phosphorylation of TrkB at tyrosine residue 705 (pTrkB Y705), a marker of TrkB activation, within the first 15 min following oral administration of R13. This significant increase over pre-treatment immunoreactivity (*p* < 0.002) remained for the entire 8-h duration of the experiment (Figure 1A). In contrast, using similar analyses, immunoreactivity to phosphorylated AKT (protein kinase B), a downstream effector of the activated phosphoinositide 3-kinase (PI3K) signaling pathway, was only transiently increased in R13-treated mice (Figure 1B). A statistically significant increase relative to pre-treatment immunoreactivity (*t*_6_ = 2.670, *p* = 0.037) was found only at the earliest post-application time (15 min), but not at later times. A single oral treatment with R13 resulted in a slightly slower (after 30 min) increase (*t*_6_ = 2.712, *p* = 0.035) in phosphorylation of mitogen-activated protein kinase (MAPK/Erk1/2) (Figure 1C). This significant (*p* < 0.05) increase persisted for the 8-h duration of the experiment. No visible change in relative immunoreactivity to pTrkB Y705, pAKT, or pMAPK/Erk1/2 was found in nerves from mice treated orally with vehicle under similar conditions (Supplementary Figure 1).

### Oral R13 Treatments Promote Successful Regeneration of Axons of More Motoneurons

**FIGURE 2:**
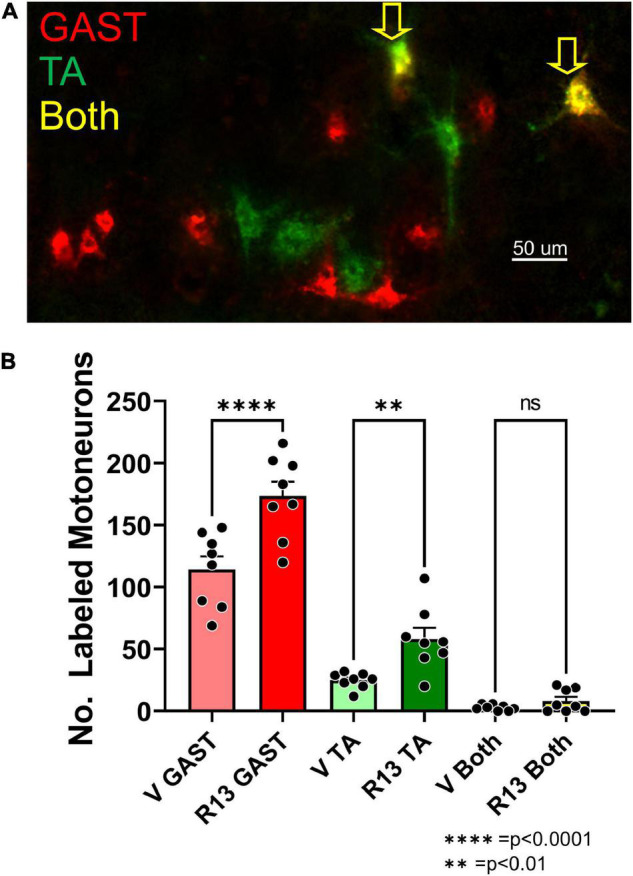
Oral R13 treatments enhance the number of motoneurons whose axons regenerated successfully. A. Image of a longitudinal section through lamina IX of the lumbar spinal cord of a mouse 4 weeks after sciatic nerve transection and repair and treatment with R13. Red and green fluorophores mark motoneurons retrogradely labeled from tracers injected into the reinnervated gastrocnemius (GAST) and tibialis anterior (TA) muscles, respectively. **(A)** Small number of motoneurons contain both labels (Both, yellow arrows). **(B)** Mean (± SEM, *n* = 8) numbers of labeled neurons in these three categories in vehicle-treated (V) and R13-treated mice. ** = p < 0.01, **** = p < 0.0001.

Motoneurons whose axons had regenerated and successfully reinnervated the two heads of GAST and the TA muscle were marked using retrograde fluorescent tracers. Examples of labeled TA (green) and GAST (red) motoneurons, and motoneurons labeled with both tracers (yellow, arrows), are shown in Figure 2A. Counts (mean ± SEM) of labeled motoneurons reinnervating these two muscles in vehicle-treated and R13-treated mice are shown in Figure 2B. Using a two-way (sex vs. treatment) ANOVA to evaluate the neuron counts from the two muscles, we found a significant difference due to treatment [*F*_(1,28)_ = 4.662, *p* = 0.040] but not sex [*F*_(1,28)_ = 0.006, *p* = 0.938] or interaction [*F*_(1,28)_ = 0.215, *p* = 0.647]. Counts from males and females were then combined and data from the different treatments compared using a one-way ANOVA. Significant differences were found [*F*_(5,42)_ = 79.15, *p* < 0.0001], and using *post hoc* (Tukey) paired testing, significantly more retrogradely labeled motoneurons were found in the R13-treated mice than in the vehicle-treated controls for both TA (*p* < 0.003) and GAST (*p* < 0.0001). For both treatment groups studied a small number of motoneurons contained both retrograde tracers, suggesting that their regenerating axons had branched and reinnervated both the GAST and TA muscles. Differences in the numbers of these doubly labeled cells between the two groups was not significant (*p* = 0.620). R13 treatments thus resulted in successful regeneration of axons of more motoneurons than vehicle-treated controls.

### Oral R13 Treatments Promote Successful Regeneration of Axons of More Sensory Neurons

**FIGURE 3:**
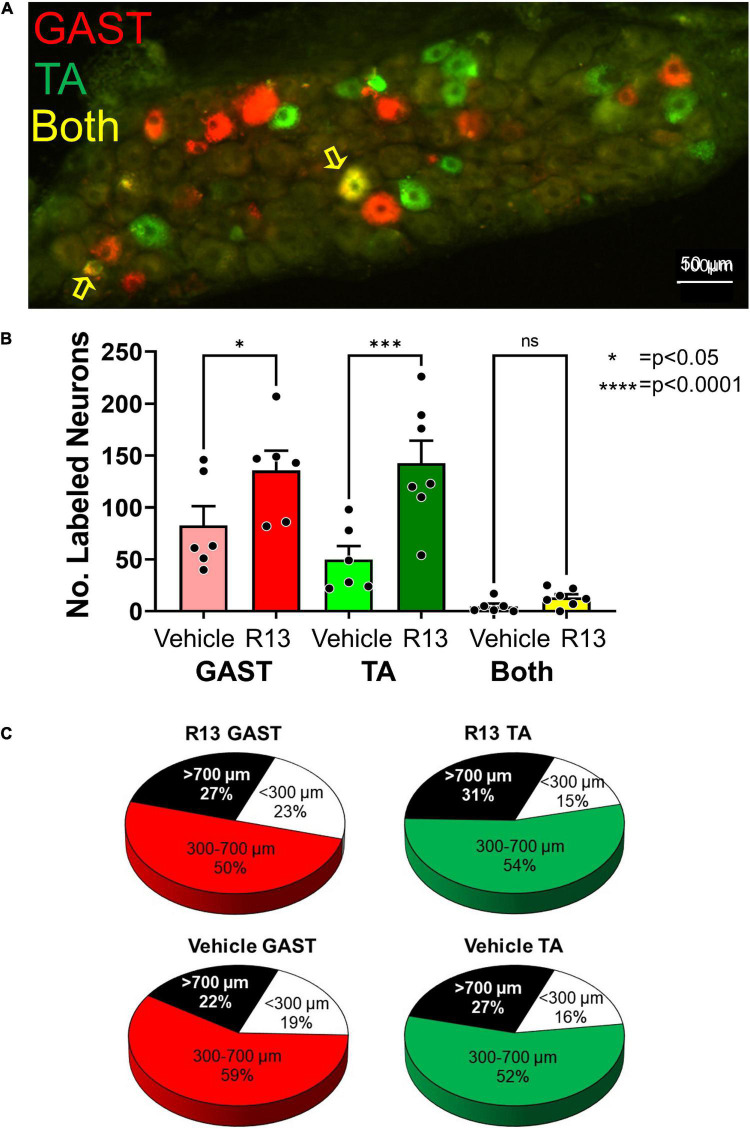
Oral R13 treatments promote regeneration of muscle afferent neurons. **(A)** Histological section through an L4 dorsal root ganglion (DRG) from a mouse treated with R13. Cell bodies of primary afferent neurons successfully reinnervating the gastrocnemius (GAST, red) or tibialis anterior (TA, green) muscles 4 weeks after sciatic nerve transection and repair are shown. Sensory neurons labeled by both tracers (yellow) are indicated by the yellow arrows. **(B)** Counts of labeled neurons (mean ± SEM, *n* = 6) in the L4 dorsal root ganglia of vehicle-treated and R13-treated mice. **(C)** Mean proportions of labeled DRG neurons in different size classes in the two different treatment groups. No significant differences were found between R13-treated and vehicle-treated mice. * = *p* < 0.05, **** = *p* < 0.0001.

We counted retrogradely labeled neurons in histological sections of the L4 DRGs (Figure 3A) 4 weeks after sciatic nerve transection and repair. Using a two-way (sex vs. treatment) ANOVA to evaluate the data from counts of sensory neurons retrogradely labeled from the two muscles together, we found a significant difference due to treatment [*F*_(1,23)_ = 21.37, *p* < 0.0001] but not sex [*F*_(1,23)_ = 0.037, *p* = 0.845] or interaction [*F*_(1,23)_ = 0.894, *p* = 0.354]. Counts from males and females were then combined and those from vehicle-treated (Figure 3B: light bars) and R13-treated (Figure 3B: dark bars) mice were compared using a one-way ANOVA. Significant differences were found [*F*_(5,32)_ = 15.56, *p* < 0.0001], and using *post hoc* paired testing, significant differences were found between R13-treated and vehicle-treated groups for both TA (*p* < 0.0001) and GAST (*p* < 0.023). No significant differences were found between these two groups for the small number of DRG neurons that were doubly labeled from tracer injections in both TA and GAST. Thus, R13 treatments resulted in the successful regeneration of more muscle sensory axons than controls.

We next evaluated the proportion of labeled DRG cells in three different size groupings (Ruscheweyh et al., 2007). We approached this size-based separation as a convenient way to compare the extent of success of axon regeneration among different groups of DRG neurons, not to distinguish between neurons of different functional classes. We expressed the number of small (<300 μm^2^), medium-sized (300–700 μm^2^), and large (> 700 μm^2^) neurons as a function of the total number of labeled neurons in the two treatment groups. Mean proportions of all DRG neurons labeled from GAST and TA in these size classes are shown in Figure 3C. We compared these proportions using a one-way ANOVA [*F*_(5,24)_ = 8.402 *p* = 0.0001]. Significantly (*p* < 0.001) more medium-sized labeled neurons were found in both treatment groups from both muscles than either small or large neurons. However, no significant differences were found in the proportions of these sensory neurons in each of the size classes between R13-treated and vehicle-treated mice for both TA and GAST.

### Oral R13 Treatments Result in Greater Muscle Reinnervation Than Controls

**FIGURE 4:**
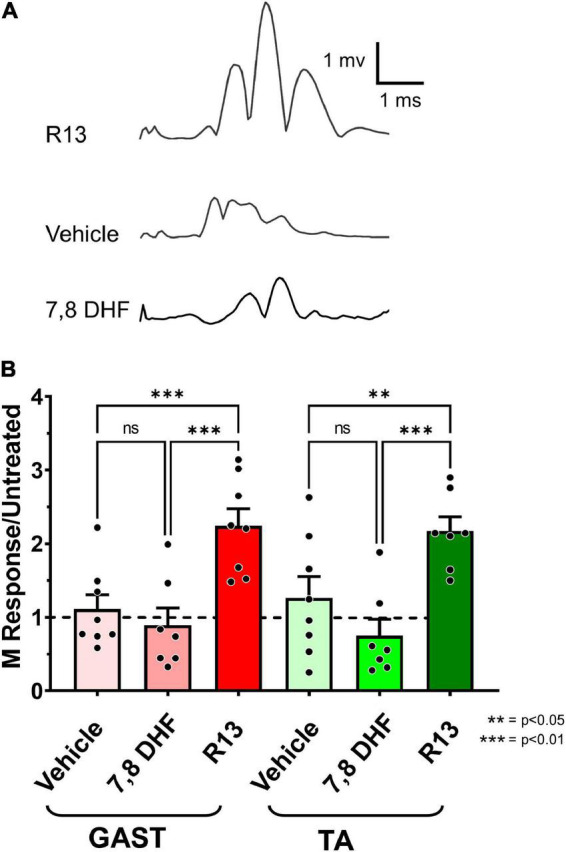
**(A)** Direct muscle (M) responses recorded from gastrocnemius muscles in response to sciatic nerve stimulation are shown 4 weeks after sciatic nerve transection and repair from a mouse treated with oral R13 (upper trace), from a mouse treated with vehicle (middle trace), and from a mouse treated with oral 7,8-DHF (lower trace). **(B)** Mean (± SEM) scaled full wave rectified M response amplitudes recorded from gastrocnemius (GAST) and tibialis anterior (TA) muscles 4 weeks after sciatic nerve transection and repair and oral treatment with vehicle (*n* = 8), 7,8-DHF (*n* = 7), or R13 (*n* = 7). The dashed horizontal line at unity represents the mean Mmax amplitude recorded from untreated mice 4 weeks after transection and repair of the sciatic nerve (Park et al., 2019). **p < 0.01, ***p < 0.001.

To evaluate the effects of R13 treatments on functional motor recovery after sciatic nerve transection and repair, we evoked direct muscle EMG responses (M responses) to sciatic nerve stimulation in GAST and TA muscles 4 weeks after injury. Examples of M responses recorded at that time from an R13-treated mouse, a vehicle-treated mouse, and a mouse treated with oral 7,8 DHF are shown in Figure 4A. The amplitudes of full wave rectified Mmax responses in the R13-treated, vehicle-treated, and oral 7,8-DHF-treated mice were scaled to the mean Mmax amplitude recorded from a series of untreated mice 4 weeks after sciatic nerve transection and repair (Park et al., 2019). Differences in these scaled Mmax amplitudes were first compared between male and female mice treated either with R13 or vehicle or 7,8-DHF (*N* = 4 of each sex in each group) using a two-way (sex and treatment) ANOVA. A significant effect of treatment [*F*_(1,12)_ = 6.775, *p* = 0.023 for GAST, *F*_(1,12)_ = 4.973, *p* = 0.04 for TA] was found, but not for sex or for interaction, so that data from the sexes were combined. Significance of differences between treatment groups was determined using a one-way ANOVA. Significant differences were found for both GAST [*F*_(2,19)_ = 12.01, *p* = 0.0004] and TA [*F*_(2,19)_ = 8.892, *p* = 0.019]. Using *post hoc* paired testing, scaled Mmax amplitudes from both muscles were found to be significantly greater in the R13-treated mice than in either the vehicle-treated mice (*p* < 0.002 for GAST, *p* < 0.010 for TA) or the mice treated with oral 7,8-DHF (*p* < 0.001 for GAST, *p* < 0.002 for TA) (Figure 4B). The scaled Mmax responses in vehicle-treated mice and mice treated with oral 7,8-DHF were not significantly different from each other, and their mean scaled Mmax amplitudes were not significantly different from unity (mean + 95% confidence interval > 1.0), indicating that among these treatments, only oral administration of R13 produced a significant enhancing effect on functional muscle reinnervation.

## Discussion

Poor functional recovery from peripheral nerve injuries is a significant clinical problem. Even though axons in peripheral nerves are capable of regenerating after injury, very few nerve injury patients experience significant functional recovery (Portincasa et al., 2007; Scholz et al., 2009). The most common reason given for these poor outcomes is the slow and inefficient process of axon regeneration. Novel treatments are required if this situation is to be improved. The present study reports the initial results of examination of one such novel experimental approach, oral administration of the prodrug R13 following sciatic nerve transection and repair.

We and others have argued that successful axon regeneration following PNI could be improved by increasing the activation of the TrkB receptor by BDNF, by either direct application of recombinant human BDNF (rhBDNF), or by activity-dependent treatments such as brief 20 Hz electrical stimulation or treadmill exercise (Gordon and English, 2016). Systemic injections of the small molecule TrkB ligands deoxygedunin or 7,8-DHF also proved effective (English et al., 2013). However, the bioavailability of rhBDNF and even 7,8-DHF is limited (Chen et al., 2018) so that successful enhancement of axon regeneration over distances like those often encountered in human patients could require multiple daily injections or/and high doses that could contribute to unwanted side effects or even toxicity. The development of the R13 prodrug sought to circumvent these limitations. It is metabolized in the liver, releasing 7,8-DHF into the systemic circulation gradually, thereby prolonging the bioavailability of that BDNF mimetic (Chen et al., 2018).

We show here that oral administration of R13 following sciatic nerve transection and repair results in a rapid and prolonged increase of TrkB phosphorylation at the site of the nerve injury. Phosphorylation of TrkB at tyrosine residue 705 is considered a critical step in TrkB receptor activation, which further regulates the phosphorylation of other TrkB positions leading to activation of downstream signaling pathways (Huang and Reichardt, 2003; Huang and McNamara, 2010). Increased BDNF and TrkB expression at the site of nerve injury has been reported (Funakoshi, 1993) and it might be presumed, therefore, that some activation of TrkB might be found. Indeed, we did find modest immunoreactivity to pTrkB Y705, relative to full length TrkB, in cut nerves prior to administration of either R13 or vehicle. However, an increase in relative pTrkB Y705 immunoreactivity was noted later only after R13 administration. We also found that R13, but not vehicle administration produced prolonged activation of pMAPK/Erk1/2, a notable downstream effector of TrkB activation. We found modest immunoreactivity for pMAPK/Erk1/2 in cut nerves prior to either R13 or vehicle administration. This observation is consistent with the findings of others in the proximal and distal segments of cut nerves (Sheu et al., 2000), where it has been implicated in the formation of repair Schwann cells (Harrisingh et al., 2004). Because Schwann cells express only the truncated form of TrkB (Funakoshi, 1993), we suggest that our finding of increased pTrkB Y705 and pMAPK/Erk1/2 activity after R13 treatments are related to signaling in other cell types, including neurons.

Consistent with its effects on TrkB signaling, oral treatment with R13 enhanced axon regeneration. The number of both motoneurons and muscle sensory neurons whose axons had regenerated and successfully reinnervated two target muscles was increased with R13 treatments. In addition, after oral treatment with R13, the mean number of motoneurons labeled by retrograde tracers injected into these muscles was within the confidence limits of those reported for intact mice (Xu et al., 2021), suggesting that oral R13 treatments produced a near complete regeneration of motor axons in as little as 4 weeks. Similarly, the restoration of muscle innervation was enhanced by R13 treatments. Amplitudes of M responses in R13-treated mice were increased 2-3-fold in R13-treated animals over both vehicle-treated controls and mice treated with oral dosing of 7,8-DHF.

The number of muscle sensory neurons labeled after tracer injections into the GAST and TA muscles also increased after R13 treatments. Similar increases in small, medium, and large neurons were noted, suggesting that the improved axon regeneration provided by increased availability of the TrkB ligand in R13-treated mice was not restricted to a particular size class. It is known that after PNI, the expression of TrkB in DRG neurons is expanded to include neurons of all size classes if those neurons are activated by electrical stimulation (English et al., 2007; Geremia et al., 2007), a treatment known to increase BDNF release (Gordon and English, 2016). We speculate that our R13 treatments improved regeneration of these different sizes of muscle sensory axons via a similar expansion of TrkB expression. Whether R13 treatments also promote the accurate reinnervation of muscle proprioceptive end organs like muscle spindles, as well as improved reinnervation of non-muscle sensory targets such as skin, awaits further investigation.

We treated mice with R13 multiple times after the nerve injury to be comparable to the application of moderate daily exercise that we have used successfully in previous studies (English et al., 2011; Sabatier and English, 2015; Gordon and English, 2016) and that also relies on increased BDNF-TrkB signaling for its effectiveness (Wilhelm et al., 2012). Our findings at 4 weeks after injury are that the numbers of successfully regenerating motor axons and the amplitudes of M responses are similar to what we have reported in mice treated with exercise. We believe that the effectiveness of our experimental R13 therapy is the result of the gradual release of the BDNF mimetic 7,8-DHF over the 2-week treatment period. However, unlike BDNF, 7,8-DHF does not bind to and signal through the common neurotrophin receptor, p75*^NTR^* (Wurzelmann et al., 2017). It is thus possible that our R13 treatments were so effective also because they avoided any anti-growth effects on regenerating axons resulting from p75*^NTR^* binding (Boyd and Gordon, 2001; McGregor et al., 2021).

## Conclusion

In conclusion, R13 treatments show considerable promise as a novel approach to enhancing functional recovery following PNI. The convenience of its oral administration, its prolonged biological activity at the site of injury, and its stimulation of both sensory and motor axon regeneration make it an experimental therapy worthy of further consideration.

## Data Availability Statement

The raw data supporting the conclusions of this article will be made available by the authors, without undue reservation.

## Ethics Statement

The animal study was reviewed and approved by the Institutional Animal Care and Use Committee of Emory University.

## Author Contributions

AE conceived the study, supervised the experiments, analyzed the data, and wrote the manuscript. DC, RI, and SSK performed the experiments, analyzed the data, and commented on the manuscript. DH assisted in the experiments, analyzed EMG data, and commented on the manuscript. SK assisted in the experiments, analyzed neuroanatomical data, and commented on the manuscript. XL performed immunoblot experiments, analyzed the data, and commented on the manuscript. KY conceived study, provided R13 reagents, and commented on the manuscript. All authors contributed to the article and approved the submitted version.

## Conflict of Interest

The authors declare that the research was conducted in the absence of any commercial or financial relationships that could be construed as a potential conflict of interest.

## Publisher’s Note

All claims expressed in this article are solely those of the authors and do not necessarily represent those of their affiliated organizations, or those of the publisher, the editors and the reviewers. Any product that may be evaluated in this article, or claim that may be made by its manufacturer, is not guaranteed or endorsed by the publisher.

## Supplementary Material

Supplementary Figure 1 Immunoblots of extracts of cut and repaired sciatic nerves from mice that had been administered the R13 vehicle (5% DMSO/0.5% methylcellulose) orally. The different lanes are from nerves collected at different times (minutes) after administration. Administration of the vehicle produced no notable change in TrkB activation (pTrkB Y705) or in either of the downstream effectors, AKT or MAPK/Erk1/2.
