# Supplementary figures and images for: Oral Treatments With the TrkB Ligand Prodrug, R13, Promote Enhanced Axon Regeneration Following Peripheral Nerve Injury
Source: Front Cell Neurosci. 2022 Apr 15;16:857664. doi: 10.3389/fncel.2022.857664 (PMC9051483; doi:10.3389/fncel.2022.857664)

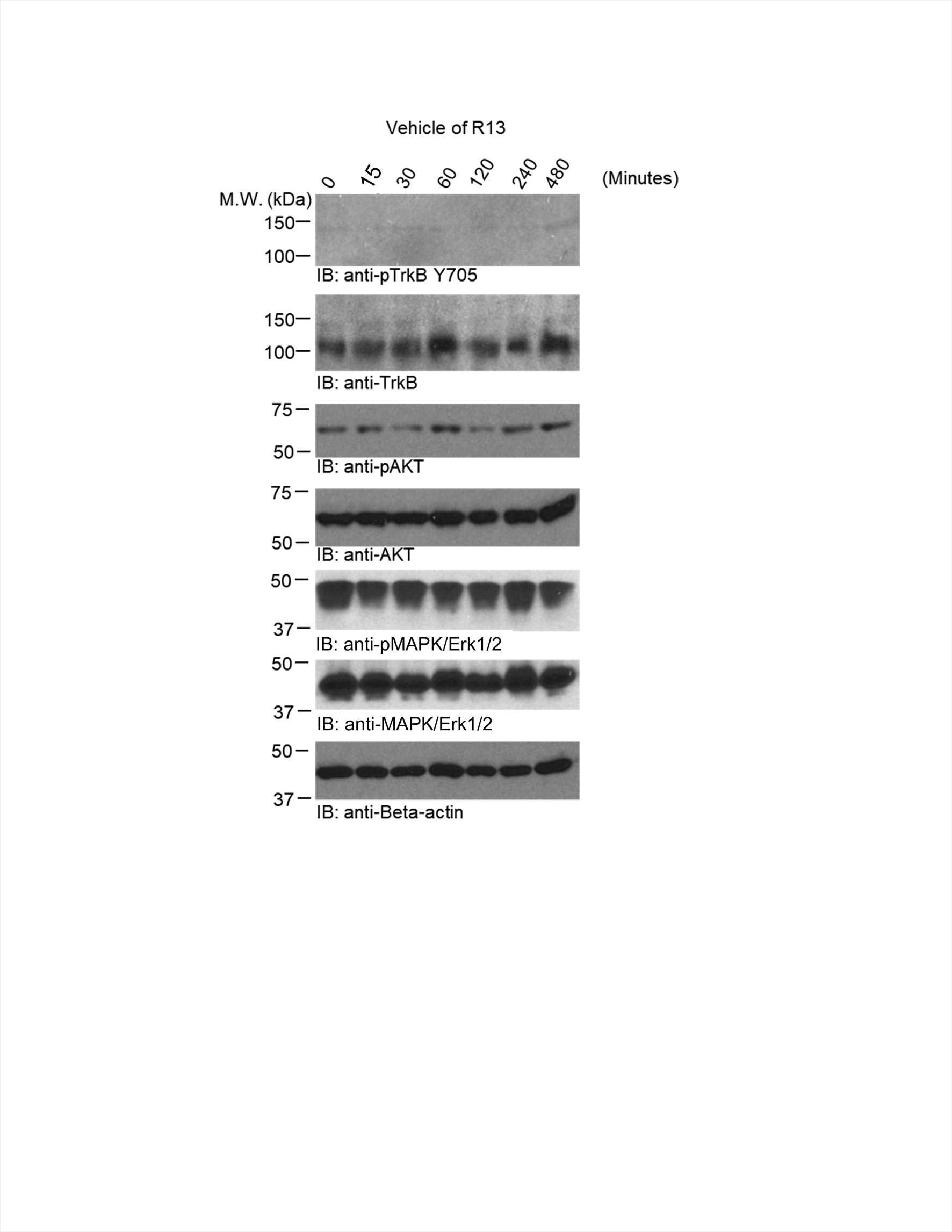

Supplement: Supplementary Figure 1 — Immunoblots of extracts of cut and repaired sciatic nerves from mice that had been administered the R13 vehicle (5% DMSO/0.5% methylcellulose) orally. The different lanes are from nerves collected at different times (minutes) after administration. Administration of the vehicle produced no notable change in TrkB activation (pTrkB Y705) or in either of the downstream effectors, AKT or MAPK/Erk1/2. [file Image_1.tif]
